# Multi-locus stepwise regression: a haplotype-based algorithm for finding genetic associations applied to atopic dermatitis

**DOI:** 10.1186/1471-2350-13-8

**Published:** 2012-01-27

**Authors:** Sven Knüppel, Jorge Esparza-Gordillo, Ingo Marenholz, Hermann-Georg Holzhütter, Anja Bauerfeind, Andreas Ruether, Stephan Weidinger, Young-Ae Lee, Klaus Rohde

**Affiliations:** 1 Max Delbrück Center for Molecular Medicine Berlin-Buch, Berlin, Germany; 2 Pediatric Pneumology and Immunology, Charité Universitätsmedizin Berlin, Berlin, Germany; 3 Institute of Biochemistry, Charité Universitätsmedizin Berlin, Berlin, Germany; 4 Institute for Clinical Molecular Biology, Christian-Albrechts-University, Kiel, Germany; 5 Department of Dermatology and Allergy, Technische Universität München, Munich, Germany; 6 Division of Environmental Dermatology and Allergy, Helmholtz Zentrum Munich and ZAUM-Center for Allergy and Environment, Technische Universität München, Munich, Germany; 7 German Institute of Human Nutrition Potsdam-Rehbrücke, Department of Epidemiology, Nuthetal, Germany

## Abstract

**Background:**

Genome-wide association studies (GWAS) provide an increasing number of single nucleotide polymorphisms (SNPs) associated with diseases. Our aim is to exploit those closely spaced SNPs in candidate regions for a deeper analysis of association beyond single SNP analysis, combining the classical stepwise regression approach with haplotype analysis to identify risk haplotypes for complex diseases.

**Methods:**

Our proposed multi-locus stepwise regression starts with an evaluation of all pair-wise SNP combinations and then extends each SNP combination stepwise by one SNP from the region, carrying out haplotype regression in each step. The best associated haplotype patterns are kept for the next step and must be corrected for multiple testing at the end. These haplotypes should also be replicated in an independent data set. We applied the method to a region of 259 SNPs from the epidermal differentiation complex (EDC) on chromosome 1q21 of a German GWAS using a case control set (1,914 individuals) and to 268 families with at least two affected children as replication.

**Results:**

A 4-SNP haplotype pattern with high statistical significance in the case control set (p = 4.13 × 10^-7 ^after Bonferroni correction) could be identified which remained significant in the family set after Bonferroni correction (p = 0.0398). Further analysis revealed that this pattern reflects mainly the effect of the well-known *FLG *gene; however, a *FLG*-independent haplotype in case control set (OR = 1.71, 95% CI: 1.32-2.23, p = 5.6 × 10^-5^) and family set (OR = 1.68, 95% CI: 1.18-2.38, p = 2.19 × 10^-3^) could be found in addition.

**Conclusion:**

Our approach is a useful tool for finding allele combinations associated with diseases beyond single SNP analysis in chromosomal candidate regions.

## Background

Single marker association analysis has been widely used to identify genetic risk factors involved in the genetics of complex diseases [1]. Previous studies have suggested that haplotypes, a collection of ordered markers along a chromosome, may be more appropriate as a unit for statistical analysis than single genetic markers [2,3]. As demonstrated by simulation studies, statistical approaches based on haplotypes can be a powerful method to characterize the genetic background of complex diseases [1,4-6]. However, since haplotypes are often not directly observable we have to use unphased genotypes to estimate haplotypes.

The advent of the gene chip technology has resulted in a multitude of genome-wide association studies (GWAS). These are in general based upon large numbers of single nucleotide polymorphisms (SNPs) genotyped along the genome for large numbers of individuals. Due to the multitude of tests along the genome, only substantial single locus associations withstand the Bonferroni correction. Another possibility to exploit these high quality data of closely spaced SNPs is to concentrate on certain candidate regions and use these closely spaced SNPs for haplotype estimation. Thus, a more thorough association analysis of genetic traits can be performed.

Using haplotypes instead of single SNPs, one has to deal with a much higher number of haplotypes than single SNPs in a candidate region. This is because k single SNPs may give rise to 2^k^-1 different SNP combinations. To overcome this issue we used stepwise regression to find (suboptimal) haplotype patterns from unphased multi-locus SNP genotype data.

Statistical methods using a given set of unphased genotype data to test haplotype effects in the framework of general linear models have already been proposed [2,3]. We combined these methods with the strategy of classical stepwise variable selection [7]. The aim of our multi-locus stepwise regression (MSR) was to find the best haplotype patterns associated with disease phenotypes.

Stepwise regression based on a limited number of SNPs to detect haplotype effects has been proposed for independent individuals [8-10] as well as for family data [11]. Some methods store in each step all haplotypes below a certain p value for extension [8,9] or select the optimal number of SNPs to combine them in a two-step approach using cross-validation [10]. In order to deal with a larger number of SNPs in candidate regions given for instance in the case of GWAS data, our method aims at the extension and outcome of a preset number of best haplotypes by using a different search strategy for case-control or family data, respectively, which have to be corrected for multiple testing and should be confirmed in an independent data set.

As proof of principle we applied our method to find haplotype patterns with effects on atopic dermatitis. As input, we utilized SNPs from the epidermal differentiation complex (EDC) located in a 1.9 Mb region on chromosome 1q21; these SNPs had been genotyped in 939 cases and 975 controls as part of a recently performed German genome-wide association study on atopic dermatitis. Findings were replicated in an independent data set of 268 complete nuclear families with at least two affected siblings affected by atopic dermatitis.

We chose the EDC region for this analysis, because it contains a lot of candidate genes for atopic dermatitis encoding structural proteins that are expressed during terminal differentiation of the human epidermis [12]. Moreover, the EDC contains the *filaggrin *gene (*FLG*), mutations in which are well-known atopic dermatitis susceptibility factors [12-15]. Linkage [16] and single marker association results [12] indicate that there may exist additional susceptibility factors beyond the known *FLG *mutations in the EDC.

## Methods

According to our case control and family data we used two different routines for the haplotype estimation and testing part of the multi-locus stepwise regression (MSR). Haplotypes and their frequencies were inferred by the expectation-maximization (EM) algorithm in the case control set [17] and by a modified EM algorithm for nuclear families [18,19].

Missing genotypes were inferred while estimating haplotypes for every individual before statistical analysis. Every haplotype association test was based on the same number of individuals; however, the number of estimated haplotypes may differ between the tests.

### Multi-locus stepwise regression (MSR)

All possible SNP pairs are tested separately by haplotype regression using a global test statistic. Details of the haplotype regression are described below. Next, the results were sorted by global p value and the first *nt *haplotype patterns were selected, where *nt *is the number of stored test results for further processing in the next step. These haplotype patterns are called "best" haplotype patterns.

As next step the remaining SNPs from the whole region were added one by one to the "best" haplotype patterns and again subjected to haplotype regression. The results were then sorted by p value and the first *nt *haplotype patterns were selected. This step was repeated until no more SNPs could be added or having achieved a preset maximum number of SNPs, or a stop criterion has been met.

### Independent individuals

In case of independent individuals haplotypes were estimated using an expectation- maximization (EM) algorithm [17]. To account for the uncertainty of the haplotype pair configuration, we weighted each haplotype pair, compatible with the genotype of an individual, by its estimated probability and applied haplotype logistic regression to the binary phenotype [17]. As global test statistic for haplotype patterns, which we used as regression model in the MSR, we applied the Likelihood ratio test, comparing the full model depending on all estimated individual haplotype probabilities with the reduced model including only the intercept. To consider further variables like sex and age the Likelihood ratio test could be extended by adding the additional variables to the full and reduced model. The most frequent haplotype was chosen as baseline. Haplotypes with an estimated frequency of less than 5% were declared as "rare", and were pooled. If all estimated haplotypes were "rare" no regression model was applicable and we skipped this haplotype pattern. To avoid singularity, quasi-complete and complete separation due to low frequent haplotypes, which is a sparse data problem and makes the regression model impracticable, we skipped the pooled rare haplotype if its frequency was less than 5%. In this case the overall p value has to be interpreted relative to the baseline and the excluded rare haplotypes.

### Nuclear families

In order to estimate haplotypes for nuclear families we used a modification of the EM algorithm for independent parents, taking into account only those haplotype pair configurations, which are compatible with the children [18,19]. For each haplotype pair configuration with its probability, the respective haplotype pair configuration of the children and the maternal or paternal origin of the haplotypes were inferred. The estimated haplotypes were considered as alleles of a multi-locus marker, and the TDT statistic for each haplotype pair configuration of a nuclear family was then calculated according to [20], taking into account its weight, as given by the product of the estimated frequencies of the respective haplotypes in the parents.

### Real data example: Application to atopic dermatitis

Atopic dermatitis is a chronic inflammatory skin disease with complex etiology, which is assumed to be influenced by multiple genetic as well as environmental factors. Gene effects on the epidermal differentiation complex (EDC) on chromosome 1q21 have already been found [21,22]. However, the roles of all genes and their combination in this region are not fully understood. The present study searches for significant haplotype patterns in the EDC region including 259 high-quality SNPs (Chromosome 1: 150,075,690-152,014,240 bp). We selected 94 tagSNPs covering all 259 SNPs by a linkage disequilibrium (LD) criterion r^2 ^> 0.8, this way suppressing uninformative SNPs in high LD. The haplotype estimation via EM algorithm does not require LD between loci under consideration, which could not be expected from the tagSNPs. In this sense an estimated haplotype is a combination of alleles at their loci, not necessarily in LD. However, since all loci lie in a restricted region on a chromosome, each individual carries at its chromosomes a pair of such combinations with its probability. This forms then a pair of physically extended haplotypes over the region as basis for multi-locus stepwise regression.

The discovery and replication data sets included in this study correspond to data sets 1 and 2, respectively, previously reported in a German genome-wide association study [12]. The discovery set included 939 unrelated German individuals with atopic dermatitis and 975 German controls. The replication data set consisted of 268 complete nuclear families comprising 1,097 individuals and 529 children with atopic dermatitis. In both study groups, the physican's diagnosis of atopic dermatitis was made according to standard criteria. More details of study design are described elsewhere [12].

We applied the MSR to the discovery set as long as the decrease of the arithmetic mean of the ten best p values' decadic logarithm in each step was greater 10 percent, as empirical stop criterion. The best 300 haplotype patterns derived by MSR in the case control set were tested in the replication family set. The results of the weighted TDT statistic were Bonferroni corrected by multiplying the TDT p value by 300 (the number of patterns from the discovery set tested in the family data).

The crucial step of the analysis is the identification of the best haplotype patterns in the discovery set. These best patterns are the result of a search strategy over the total number of patterns possible and represent only a suboptimal solution. They may be corrected, however, for search strategy and multiple testing, taking the total number of possible of patterns ($\overset{\text{m}}{\underset{\text{i}=1}{\sum }}\left(\begin{array}{c} \text{n}\\ \text{i} \end{array}\right)$ with n as number of SNPs in the region and m as the number of loci included in selected best patterns) as Bonferroni correction.

The analysis was carried out using the statistical software R (version 2.11.1) [23]. To identify tagSNPs we used Tagger [24] implemented in Haploview 4.2 [25]. Information on SNP location was taken from the database SNPselector (NCBI assembly 36, dbSNP build 126) [26]. All p values lower than 0.05 were considered as statistically significant.

## Results

A total of 1,914 individuals (939 individuals with atopic dermatitis and 975 controls) and 268 nuclear families (536 parents and 529 affected children) were included in the present study. The selected 259 high-quality autosomal SNPs from the epidermal differentiation complex (EDC) are located on chromosome 1q21 (150,075,690-152,014,240 kb). We identified 94 tagSNPs using a linkage disequilibrium (LD) criterion r^2 ^> 0.8 for compressing the genotype information, avoiding haplotype patterns containing the same information through high LD, and minimizing computational time.

Running the MSR over the 94 tagSNPs in the discovery set adding one SNP at a time to the interim best haplotype patterns led to a decrease of p values. The mean of the best ten p values decreased from 6.5 × 10^-7 ^(2 loci) over 3.07 × 10^-13 ^(4 loci) to 1.04 × 10^-13 ^(5 loci). The results of the 10 best SNP patterns in each step of the MSR can be found in Additional File 1, Table S1. All haplotype patterns in all steps came out with a nominal p value lower than 0.001. The change of the mean value of the best ten -log10(p values) decreased from 56% for step from 2 to 3 loci to 4% for step 4 to 5 loci. Stopping at a percentage change lower than 10% meant that we stopped MSR at patterns of 4 SNPs.

Result of the MSR search over the discovery set is a list of 300 best 4-SNP haplotype patterns with nominal p values in a range from 3.74 × 10^-14 ^to 1.19 × 10^-9^. It is to be noticed that all of these withstand the Bonferroni correction ($\overset{4}{\underset{\text{i}=1}{\sum }}\left(\begin{array}{c} 94\\ \text{i} \end{array}\right)=3188010$ possible patterns).

To replicate our findings the 300 best 4-SNP haplotype patterns derived by MSR in the discovery set were subsequently tested for association in the family set (Table 1). After Bonferroni correction of the p values of the TDT-statistic, one haplotype pattern showed a corrected p value with 0.03976 (nominal 0.00013), lower than the arbitrary significance threshold of 0.05. This best replicated haplotype pattern contained the SNPs rs7550106 (*HRNR*), rs499697 (*LCE3C*), rs17659389 (*LCE3C*), and rs17670505 (*LCE1C*).

**Table 1:** Resulting ten best haplotype patterns after replication of the multi-locus stepwise regression.

No.	SNP 1	SNP 2	SNP 3	SNP 4	MSR p value	MSR p value (corrected)	TDT p value	TDT p value (corrected)
**1**	**rs7550106**	**rs499697**	**rs17659389**	**rs17670505**	**1.30 × 10**^**-13**^	**4.13 × 10**^**-7**^	**0.00013**	**0.03976**
2	rs499697	rs17670505	rs576941	rs16835086	2.99 × 10^-10^	9.54 × 10^-4^	0.00033	0.09809
3	rs13373771	rs499697	rs6702463	rs17670505	9.85 × 10^-10^	3.14 × 10^-3^	0.00034	0.10260
4	rs6678672	rs4845766	rs499697	rs17659389	9.70 × 10^-10^	3.10 × 10^-3^	0.00040	0.11999
5	rs6678672	rs4845766	rs13373771	rs17659389	1.05 × 10^-09^	3.34 × 10^-3^	0.00051	0.15153
6	rs7550106	rs499697	rs989834	rs17670505	4.20 × 10^-10^	1.34 × 10^-3^	0.00053	0.15796
7	rs11204897	rs13373771	rs6701221	rs17670505	2.51 × 10^-10^	8.01 × 10^-4^	0.00055	0.16504
8	rs499697	rs17659389	rs17670505	rs16835086	4.77 × 10^-10^	1.52 × 10^-3^	0.00058	0.17369
9	rs2999547	rs499697	rs17659389	rs17670505	1.65 × 10^-10^	5.25 × 10^-4^	0.00061	0.18254
10	rs13373771	rs1923508	rs6701221	rs17670505	7.36 × 10^-10^	2.35 × 10^-3^	0.00074	0.22309

Multi-locus stepwise regression (MSR p value) was used for case control set (n = 1,914) and replicated in family set (268 families) using the weighted TDT statistic. Bonferroni corrections were done by multiplying 3188010 to the MSR p value and 300 to the TDT p value. Only the first haplotype pattern kept significant after Bonferroni correction.

Since longer range LD with *filaggrin *(*FLG*) mutations has been previously described in the EDC region and, especially, SNP rs7550106 of the best haplotype pattern is in the same haplotype block as *FLG *gene, we tried to find out whether the haplotype association derived was due to an underlying association with the four known *FLG *gene mutations 2282del4, R501X, R2447X, and S3247X. In order to analyze this in more detail, we removed all individuals carrying any of these *FLG *mutations from the data sets and tested whether the best haplotype pattern still revealed significant association. In the *FLG *reduced set, after exclusion of 309 individuals (240 cases and 69 controls) from the case control set and 106 families from the family set, the global p values were still significant and changed from 1.30 × 10^-13 ^(full case control set) to 0.00015 (*FLG *reduced case control set) and from 0.00013 (full family set) to 0.0151 (*FLG *reduced family set), indicating an effect additional to the four *FLG *mutations.

Another way to evaluate the *FLG *mutation effects is the analysis of 8-SNP haplotypes, which were built independent of the physical position, by the four SNPs of the best haplotype pattern in front followed by the four *FLG *mutation loci (Table 2). An LD plot of the physically ordered SNPs can be found in Additional File 1, Figure S1. We analyzed the 8-SNP haplotypes in the complete and the *FLG *reduced data sets, separately. In order to study low-frequent haplotypes due to the low frequencies of the *FLG *mutations we had to keep haplotypes with a frequency down to 0.001, instead of the generally used 0.05. The minor SNP allele was coded as 1 and the major one as 2.

**Table 2:** 8-SNP haplotype association tests for the best haplotype pattern (rs7550106, rs499697, rs17659389, rs17670505) derived by multi-locus stepwise regression (MSR) with four known *FLG *mutations (S3247X, R2447X, 2282del4, R501X) added in that order for case control set and replicated by family set.

		Case control set						Family set						
		
		Freq. (n = 1914)		FLG (n = 1914)		NON-FLG (n = 1605)		FLG (268 families)				NON-FLG (162 families)		
		
Haplotypes		Cases	Controls	OR	p value	OR	p value	Freq.	T:U	OR	p value	T:U	OR	p value
2222	2222	0.3656	0.4028	0.83	1.05 E-02	1.07	4.10E-01	0.3992	206.3:256.1	0.81	2.08 E-02	138.6:152.4	0.91	4.18 E-01
2222	2212	0.0102	0.0035	4.92	2.67 E-03			0.0039	2.0:6.0	0.33	1.58 E-01			
2222	2221	0.0052	0.0062	0.80	6.50 E-01			0.0011	2.1:0.1	38.89	1.66 E-01			
2222	2122	0.0030	0.0041	0.63	4.80 E-01			NA	NA	NA	NA			

**2222**	**Total**	**0.3841**	**0.4166**	**0.85**	**2.43 E-02**			**0.4042**	**210.4:262.1**	**0.80**	**1.74 E-02**			

2212	2222	0.1180	0.1392	0.78	2.43 E-02	0.95	6.34E-01	0.1077	110.6:91.8	1.20	1.86 E-01	66.5:59.6	1.11	5.42 E-01
2212	2212	0.0014	0.0018	0.53	6.35 E-01			NA	NA	NA	NA			
2212	2221	0.0010	0.0012	0.63	7.79 E-01			0.0011	1.1:1.1	1.00	1.00 E+00			

**2212**	**Total**	**0.1204**	**0.1422**	**0.76**	**1.28 E-02**			**0.1088**	**111.7:92.9**	**1.20**	**1.89 E-01**			

2122	2222	0.1289	0.1488	0.81	4.78 E-02	0.90	3.44E-01	0.1205	93.7:126.6	0.74	2.66 E-02	64.1:79.9	0.80	1.87 E-01
2122	2221	0.0241	0.0032	12.65	1.75 E-10			0.0267	35.2:18.3	1.92	2.07 E-02			
2122	2212	0.0123	0.0037	7.00	1.21 E-04			0.0068	7.0:8.0	0.87	7.91 E-01			
2122	2122	0.0114	0.0012	22.62	7.16 E-07			0.0115	15.0:9.0	1.67	2.21 E-01			
2122	2211	0.0034	0.0000	NA	NA			NA	NA	NA	NA			

**2122**	**Total**	**0.1800**	**0.1569**	**1.23**	**2.69 E-02**			**0.1655**	**150.9:161.9**	**0.93**	**5.34 E-01**			

2121	2212	0.0448	0.0028	31.26	3.10 E-24			0.0538	74.0:38.0	1.95	6.61 E-04			
2121	2222	0.0267	0.0295	0.88	5.63 E-01	0.97	8.94E-01	0.0123	11.8:13.8	0.85	6.92 E-01	5.7:9.7	0.59	3.08 E-01
2121	2221	0.0075	0.0000	NA	NA			0.0019	2.0:2.0	1.00	1.00 E+00			

**2121**	**Total**	**0.0790**	**0.0322**	**2.65**	**5.22 E-10**			**0.0680**	**87.7:53.7**	**1.63**	**4.22 E-03**			

2112	2222	0.0562	0.0892	0.49	2.43 E-06	0.59	8.22E-04	0.0582	46.8:68.0	0.69	4.73 E-02	24.2:36.3	0.67	1.19 E-01
2112	2212	0.0041	0.0016	6.23	4.20 E-02			0.0048	4.0:6.0	0.67	5.27 E-01			
2112	2221	0.0015	0.0010	2.59	4.73 E-01			0.0029	2.1:3.0	0.69	6.75 E-01			

**2112**	**Total**	**0.0618**	**0.0917**	**0.60**	**3.89 E-04**			**0.0659**	**52.8:77.1**	**0.69**	**3.37 E-02**			

1222	2222	0.0982	0.0785	1.36	1.69 E-02	1.71	5.55E-05	0.1186	124.8:88.6	1.41	1.30 E-02	92.0:54.9	1.68	2.19 E-03
1222	1222	0.0061	0.0010	8.05	2.77 E-03			NA	NA	NA	NA			

**1222**	**Total**	**0.1043**	**0.0795**	**1.42**	**4.81 E-03**			**0.1186**	**124.8:88.6**	**1.41**	**1.30 E-02**			

Logistic regression was used for testing a single haplotype for the case control set and the weighted TDT statistic was used for a single haplotype by computing transmitted (T) versus non-transmitted (U) haplotypes. Calculations were carried out for the full set (*FLG*) and the *FLG *reduced sets (NON-*FLG*). The minor allele was coded as 1 and the major one as 2. For the sake of following the rare *FLG *mutations the frequency threshold for the 8-SNP haplotype estimation had been lowered to 0.001, however, only combined with best pattern haplotypes down to a total frequency > 0.05 in the full case set.

Abbreviations are as follows: Freq., haplotype frequency; OR, odds ratio; *FLG*, *Filaggrin *gene; T:U, transmitted:non-transmitted; NA, not available.

Here we are interested in the substructure of the best pattern, the effect of each of the 8-SNP haplotypes. Therefore, each of these haplotypes has been tested separately using the logistic regression in the case control set resulting in per haplotype odds ratios and their approximative 95% confidence intervals (95% CI). In the family set the odds ratios were calculated by the weighted number of transmitted haplotypes divided by the weighted number of non-transmitted haplotypes; the corresponding 95% CIs were calculated by the exact McNemar test using the nearest integer number of the weighted number of transmitted and non-transmitted haplotypes [27]. Though p values and 95% CIs are only nominal, the odds ratios reflect strength and direction of haplotype effects in the substructure of our best pattern.

The most frequent best pattern haplotype 2222 had in the full set a total frequency of 0.3841 in cases, 0.4166 in controls, and 0.4042 in the families. This haplotype had been split into 8-SNP haplotypes according to the *FLG *mutations. Since the *FLG *mutations are rare, the most frequent haplotype (2222-2222) contains no *FLG *mutations. The odds ratio (OR = 0.83; 95% CI: 0.72-0.96; p = 0.0105) of that haplotype was slightly significant in the full set and changed toward the null (OR = 1.07; 95% CI: 0.92-1.24; p = 0.410) losing significance, if all *FLG *carriers were excluded. This most frequent haplotype is protective, outbalancing the rare mutation effects of the *FLG *loci, e.g. the odds ratio of haplotype 2222-2212 was 4.92 (95% CI: 1.44-16.79; p = 0.00267) with a frequency of 0.0102 in cases and 0.0035 in controls.

As seen for best pattern haplotype 2222, the outbalancing protective effect of all best haplotypes patterns carrying no *FLG *mutation (xxxx-2222) is a general feature of the significant results of Table 2 caused by the opposite *FLG *mutation effects. An exception is the best pattern haplotype 1222 which showed a risk effect of the non-*FLG *haplotype (1222-2222).

Of further interest were the best pattern haplotypes 2122, 2121, 2112, and 1222, which allowed getting deeper insight into the combination of these haplotypes with *FLG *mutations. We found that single *FLG *mutations in combination with one of the best pattern haplotypes showed strong effects with high significance, as can be seen for 1222-1222 (Case control set: OR = 8.05; 95% CI: 1.51-42.85; p = 0.0028), 2122-2122 (Case control set: OR = 22.62; 95% CI: 4.17-122.73; p = 7.16 × 10^-7^; Family set: OR = 1.67; 95% CI: 0.68-4.32; p = 0.22), 2121-2212 (Case control set: OR = 31.26; 95% CI: 11.17-87.46; p = 3.10 × 10^-24^; Family set: OR = 1.95; 95% CI: 1.40-2.96; p = 0.00066), 2122-2221 (Case control set: OR = 12.65; 95% CI: 4.54-35.22; p = 1.75 × 10^-10^; Family set: OR = 1.92; 95% CI: 1.07-3.64; p = 0.0207). As expected, these effects turn to the null in the *FLG *reduced data sets, this way underlying a strong *FLG *effect reflected by the best pattern. The relatively large confidence intervals of the odds ratios in the case control set are due to the low haplotype frequencies.

Most of the haplotype effects could be explained by the four known *FLG *mutations but not all. For haplotype 1222-2222, already mentioned, with a frequency of 0.0982 in cases, 0.0785 in controls, and 0.1186 in families, containing only the risk allele of SNP rs7550106 and the major alleles of the other SNPs, we found an increase in significance and odds ratio on single haplotype basis, indicating an additional effect to the four known *FLG *mutations. It showed an increase in the odds ratio for that single haplotype from 1.36 (95% CI: 1.06-1.74; p = 0.0169) to 1.71 (95% CI: 1.32-2.23; p = 0.000056) in case control set and from 1.41 (95% CI: 1.06-1.86; p = 0.013) to 1.68 (95% CI: 1.18-2.38; p = 0.00219) in family set after exclusion of individuals carrying one or more *FLG *mutations. This indicates an additional *FLG *independent effect on atopic dermatitis.

## Discussion

A multi-locus stepwise regression (MSR) strategy has been developed to identify haplotype patterns or at least multi-locus allelic combinations with genetic effects from unphased multi-locus genotype data on phenotype. The MSR was applied to 94 tagSNPs out of 259 SNPs from the epidermal differentiation complex (EDC) on chromosome 1q21 with genotype data from a German genome-wide association study to find genetic markers associated with atopic dermatitis. We could successfully apply our method to the identification of the well-known *filaggrin *(*FLG*) mutations (2282del4, R501X, R2447X, and S3247X) and we could show an effect additional to these.

Our method focussed on forward selection due to the high number of SNPs included, which made a backward selection starting with the full number of SNPs impracticable. Thus we started with pairwise haplotypes and kept in every step the most significant 300 ones for the extension by one SNP (*nt *= 300). This restriction, to follow only 300 best patterns, is owed to computational feasibility and leads to a mere suboptimal solution. We may miss a pattern which fails to reach the best 300 patterns in an earlier step and may reach the top in later steps. To evaluate the effect of the pre-defined number of stored tests in each step (*nt*) we varied *nt *to 50, 100, and 500. For all of these selection strategies we obtained the same result for the best patterns due to the fact that our best patterns showed a strong effect in the case control set. In smaller samples or samples with a weaker genetic effect the variation of *nt *could have an influence on the final selected patterns.

The stepwise algorithm stops if a further extension of the pattern by one SNP does not lead to an essential increase in significance. We used as empirical stop criterion a decrease of the best ten -log10(p values) mean by less than 10%. Our stop criterion differs from those more elaborated ones in hapConstructor [8] and SHARE [10]. It has been chosen with the aim to estimate the general effect of stepwise added SNPs in a simple manner. Even in the unlikely case that only one of the 10 best patterns would increase by one order of magnitude and the other nine were not changed, the search would not stop. The saturation effect of our criterion with increasing numbers of SNPs for our data is presented in Additional File 1, Figure S2. However, for other data sets saturation may come up for different SNP numbers. Since the best patterns for each step are given out in an ordered list as shown in Additional File 1, Table S1, we recommend checking those lists carefully for patterns down to only two SNPs with a higher significance than the patterns in the last step.

Two other parameters influence the search: the maximum number of SNPs in the patterns (*maxSNP*) and the number of patterns kept in each step (*nt*). The maximum number of SNPs limits the maximum length the search pattern should have. Clearly, this should be larger than the onset of the saturation effect. If this is not the case, *maxSNP *should be set higher. On the other hand if maxSNP allows longer patterns the frequencies of estimated haplotypes will get smaller and smaller making a reliable statistics problematic. The number of SNP patterns kept in each step (*nt*) influences the sensitivity of our method. The larger *nt*, the deeper the search, and the better the chance to not overlook a true positive result. On the other hand, large *nt *values need more memory and computing time, so that one has to find a trade-off between sensitivity and feasibility for each data set. To simulate this process and to give an advice for setting the parameters is a difficult task and we must admit that in the light of our straightforward approach we have refrained from simulations and power calculations.

It should be clear, that p values of patterns found by MSR in a discovery set are inflated by the search process and may give only a locus pattern which (sub)-optimally differentiates between affected and unaffected individuals. Unlike hapConstructor [8] and SHARE [10] which determine the significance of the best pattern from only one data set via permutation tests, we rely on an independent data set for replication. Without such a replication set we had to carry out the lengthy procedure of permutation testing as well, or if possible, use the maximum of different patterns as "conservative" Bonferroni correction not taking into account the prior probability of the candidate region.

The restriction to use the tagSNPs of the region had a twofold effect. It reduced the required computing time by reducing the number of loci and haplotype patterns to analyze, and it avoided similar patterns, which differ only in loci with high LD, to be stored in the list of the best 300 pattern for the next step.

The best haplotype pattern found in the discovery set and replicated in an independent set of nuclear families consists of SNPs from *hornerin *(*HRNR*) and the *late cornified envelope *(*LCE*) gene cluster in the epidermal differentiation complex (EDC). These genes are involved in skin barrier function. Statistical significance of the pattern does not necessarily mean a functional effect of all its SNP on atopic dermatitis; it could be sufficient that the inclusion of the SNP information of those genes provides only a proper background for analyzing the atopic dermatitis effects best.

Using this background we tried to answer the question, whether the mutation of SNP rs7550106 located in the *FLG *block is a pure reflection of the known *FLG *effect [13]. We have two indications that there is an additional effect. Removal of all individuals carrying at least one of the known *FLG *mutations kept the found pattern significant in both data sets. Extending the best pattern found by the known *FLG *mutations, and analyzing the resulting 8-SNP haplotypes in the full set, led to highly significant haplotypes carrying a *FLG *mutation. In addition, a significant haplotype carrying none of these mutations could be found, whose effect came then out much stronger in the *FLG *reduced data sets. This finding is in line with the indication of residual linkage in the EDC region after accounting for two *FLG *mutations (R501X and 2282del4), made by [14], but is much more distinct with respect to the underlying haplotype structure.

Several studies have proposed stepwise regression based on haplotypes [8-11]. Like these our method assesses the effect of stepwise enlarged haplotypes on phenotypes of interest, applying a different search strategy for a larger number of SNPs. These approaches are a powerful tool by excluding uninformative SNPs and concentrating on the informative ones for distinction between affected and unaffected individuals, thus leading to more concise interpretations.

While the MSR worked well for our study, its function can be easily extended to other types of analysis, e.g. survival data of a cohort study. It can be applied to other phenotypes, such as continuous or categorical phenotypes.

## Conclusions

To conclude, the MSR is an effective approach to identify subsets of SNPs the haplotypes of which are significantly associated with a phenotype. The MSR keeps the advantages of stepwise regression models and haplotype statistical analysis. The MSR was, as proof of principle, successfully applied to the case control and family sets of atopic dermatitis patients and yielded one, significantly replicated, haplotype pattern including SNPs from *HRNR *and the *LCE *gene cluster. It was shown that haplotype effects independent of the four known *FLG *mutations could be detected; however, we cannot exclude a yet unknown *FLG *mutation beyond those four *FLG *mutations. Further replication through additional studies, fine mapping and functional studies will be required to gain better understanding of etiological determinants underlying this allergic disorder. The MSR provides an in-depth analysis of the influence of SNP combinations on phenotypes beyond single SNP analyses.

## Competing interests

The authors declare that they have no competing interests.

## Authors' contributions

SK and KR carried out the programming and statistical calculations. SK and KR have prepared the manuscript. JEG, IM, HGH, AB, AR, SW, and YAL gave input on the statistical analyses, interpretation of results, and drafting of the manuscript.

All authors read and approved the final manuscript.

An R package "HapEstXXR" including our method is available under CRAN http://cran.r-project.org.

## Pre-publication history

The pre-publication history for this paper can be accessed here:

http://www.biomedcentral.com/1471-2350/13/8/prepub

## Supplementary Material

Additional file 1 **Table S1. 10 best SNP patterns in each step of the Multi-locus stepwise regression (MSR) based on case control set (n = 1914)**. The results of the global test statistic (Likelihood ratio p value) and model criteria (Nagelkerke R^2^, AIC, and BIC) of the haplotype logistic regression models of the best 10 SNP patterns in each step are shown. **Figure S1**. LD plot of the four SNPs of the best 4-SNP pattern and the four FLG mutations based on the case control set. An LD plot (Haploview 4.2) shows on the basis of D' the LD-structure of the best 4-SNP pattern formed together with the known four FLG mutations in physical locus order. **Figure S2**. Mean of -log10(p values) of best 10 p values in each step of the Multi-locus stepwise regression based on case control sample. This figure shows the saturation effect of decrease of the mean of the ten best transformed p values with increasing pattern length. Click here for file
