# Supplementary material for: Multi-locus stepwise regression: a haplotype-based algorithm for finding genetic associations applied to atopic dermatitis
Source: BMC Med Genet. 2012 Jan 27;13:8. doi: 10.1186/1471-2350-13-8 (PMC3398269; doi:10.1186/1471-2350-13-8)
Supplement: Additional file 1 — Table S1. 10 best SNP patterns in each step of the Multi-locus stepwise regression (MSR) based on case control set (n = 1914). The results of the global test statistic (Likelihood ratio p value) and model criteria (Nagelkerke R2, AIC, and BIC) of the haplotype logistic regression models of the best 10 SNP patterns in each step are shown. Figure S1. LD plot of the four SNPs of the best 4-SNP pattern and the four FLG mutations based on the case control set. An LD plot (Haploview 4.2) shows on the basis of D' the LD-structure of the best 4-SNP pattern formed together with the known four FLG mutations in physical locus order. Figure S2. Mean of -log10(p values) of best 10 p values in each step of the Multi-locus stepwise regression based on case control sample. This figure shows the saturation effect of decrease of the mean of the ten best transformed p values with increasing pattern length. [file 1471-2350-13-8-S1.DOC]

**Table S1: 10 best SNP patterns in each step of the Multi-locus stepwise regression (MSR) based on case control set (n=1914)**: Results of the global test statistic (Likelihood ratio (LR) p value) and model criteria (Nagelkerke R², AIC, and BIC) of the haplotype logistic regression model. Patterns in gray allow to follow the development of our best 4-SNP pattern (dark grey)

| **SNP1** | **SNP2** | **SNP3** | **SNP4** | **SNP5** | **log likelihood** | **LR Chi2** | **LR**  **df** | **LR p value** | **Nagelkerke R²** | **AIC** | **BIC** |
| --- | --- | --- | --- | --- | --- | --- | --- | --- | --- | --- | --- |
| **2 SNPs at a time** | |  |  |  |  |  |  |  |  |  |  |
| rs499697 | rs17670505 |  |  |  | -1307.15 | 38.38 | 2 | 4.62E-09 | 0.0265 | 2620.3 | 2636.9 |
| rs13373771 | rs17659389 |  |  |  | -1306.62 | 39.46 | 3 | 1.39E-08 | 0.0272 | 2621.2 | 2643.4 |
| rs499697 | rs576941 |  |  |  | -1307.11 | 38.47 | 3 | 2.25E-08 | 0.0265 | 2622.2 | 2644.4 |
| rs499697 | rs17659389 |  |  |  | -1308.24 | 36.20 | 3 | 6.79E-08 | 0.0250 | 2624.4 | 2646.7 |
| rs13373771 | rs17670505 |  |  |  | -1310.60 | 31.50 | 2 | 1.45E-07 | 0.0218 | 2627.1 | 2643.8 |
| rs499697 | rs10888527 |  |  |  | -1310.35 | 31.99 | 3 | 5.26E-07 | 0.0221 | 2628.7 | 2650.9 |
| rs13373771 | rs6701221 |  |  |  | -1310.71 | 31.28 | 3 | 7.44E-07 | 0.0216 | 2629.4 | 2651.6 |
| rs2146114 | rs17670505 |  |  |  | -1311.17 | 30.36 | 3 | 1.16E-06 | 0.0210 | 2630.3 | 2652.5 |
| rs4240887 | rs17670505 |  |  |  | -1313.08 | 26.54 | 2 | 1.73E-06 | 0.0184 | 2632.1 | 2648.8 |
| rs499697 | rs6663448 |  |  |  | -1311.79 | 29.11 | 3 | 2.13E-06 | 0.0201 | 2631.5 | 2653.8 |
|  |  |  |  |  |  |  |  |  |  |  |  |
| **3 SNPs at a time** | |  |  |  |  |  |  |  |  |  |  |
| rs499697 | rs17659389 | rs17670505 |  |  | -1298.17 | 56.35 | 4 | 1.69E-11 | 0.0387 | 2606.3 | 2634.1 |
| rs499697 | rs17670505 | rs576941 |  |  | -1298.66 | 55.36 | 4 | 8.88E-11 | 0.0380 | 2607.3 | 2635.1 |
| rs499697 | rs989834 | rs17670505 |  |  | -1300.07 | 52.54 | 4 | 1.06E-10 | 0.0361 | 2610.1 | 2637.9 |
| rs6691350 | rs499697 | rs17670505 |  |  | -1300.30 | 52.09 | 4 | 1.32E-10 | 0.0358 | 2610.6 | 2638.3 |
| rs499697 | rs17670505 | rs6703014 |  |  | -1302.07 | 48.55 | 3 | 1.62E-10 | 0.0334 | 2612.1 | 2634.3 |
| rs7550106 | rs499697 | rs17670505 |  |  | -1300.87 | 50.94 | 4 | 2.29E-10 | 0.0350 | 2611.7 | 2639.5 |
| rs499697 | rs913998 | rs17670505 |  |  | -1301.09 | 50.51 | 4 | 2.83E-10 | 0.0347 | 2612.1 | 2639.9 |
| rs499697 | rs17670505 | rs3006423 |  |  | -1301.14 | 50.42 | 4 | 2.95E-10 | 0.0347 | 2612.2 | 2640.0 |
| rs499697 | rs17670505 | rs3891075 |  |  | -1301.28 | 50.13 | 4 | 3.40E-10 | 0.0345 | 2612.5 | 2640.3 |
| rs13373771 | rs499697 | rs576941 |  |  | -1298.94 | 54.82 | 6 | 5.05E-10 | 0.0376 | 2611.8 | 2650.7 |
|  |  |  |  |  |  |  |  |  |  |  |  |
| **4 SNPs at a time** | |  |  |  |  |  |  |  |  |  |  |
| rs499697 | rs17659389 | rs6702463 | rs17670505 |  | -1287.41 | 77.87 | 7 | 3.74E-14 | 0.0532 | 2590.8 | 2635.2 |
| rs499697 | rs17659389 | rs6701376 | rs17670505 |  | -1287.31 | 78.07 | 8 | 1.20E-13 | 0.0533 | 2592.6 | 2642.6 |
| **rs7550106** | **rs499697** | **rs17659389** | **rs17670505** |  | **-1290.13** | **72.43** | **6** | **1.30E-13** | **0.0495** | **2594.2** | **2633.1** |
| rs499697 | rs17659389 | rs11586156 | rs17670505 |  | -1287.54 | 77.61 | 8 | 1.48E-13 | 0.0530 | 2593.0 | 2643.0 |
| rs499697 | rs17659389 | rs17670505 | rs6703014 |  | -1292.21 | 68.27 | 5 | 2.35E-13 | 0.0467 | 2596.4 | 2629.7 |
| rs499697 | rs17659389 | rs17670505 | rs576941 |  | -1289.53 | 73.64 | 7 | 2.71E-13 | 0.0503 | 2595.0 | 2639.5 |
| rs13373771 | rs499697 | rs17659389 | rs17670505 |  | -1291.00 | 70.69 | 6 | 2.95E-13 | 0.0484 | 2596.0 | 2634.8 |
| rs2282302 | rs499697 | rs17670505 | rs576941 |  | -1289.82 | 73.05 | 7 | 3.57E-13 | 0.0499 | 2595.6 | 2640.1 |
| rs13373771 | rs499697 | rs17670505 | rs576941 |  | -1290.55 | 71.60 | 7 | 7.02E-13 | 0.0490 | 2597.0 | 2641.5 |
| rs13373771 | rs17659389 | rs6671975 | rs17670505 |  | -1290.66 | 71.37 | 7 | 7.80E-13 | 0.0488 | 2597.3 | 2641.7 |
|  |  |  |  |  |  |  |  |  |  |  |  |
| **5 SNPs at a time** | |  |  |  |  |  |  |  |  |  |  |
| rs13373771 | rs499697 | rs17659389 | rs17670505 | rs576941 | -1283.18 | 86.32 | 8 | 2.55E-15 | 0.0588 | 2584.3 | 2634.8 |
| rs13373771 | rs499697 | rs17659389 | rs17670505 | rs10888527 | -1285.60 | 81.49 | 8 | 2.44E-14 | 0.0556 | 2589.2 | 2639.2 |
| rs7550106 | rs499697 | rs17659389 | rs17670505 | rs6703014 | -1288.89 | 74.91 | 6 | 4.01E-14 | 0.0512 | 2591.7 | 2630.6 |
| rs499697 | rs17659389 | rs6701376 | rs17670505 | rs6703014 | -1287.85 | 76.99 | 7 | 5.65E-14 | 0.0526 | 2591.7 | 2636.1 |
| rs13373771 | rs499697 | rs17659389 | rs6702463 | rs17670505 | -1286.69 | 79.31 | 8 | 6.73E-14 | 0.0541 | 2591.3 | 2641.3 |
| rs7550106 | rs499697 | rs17659389 | rs17670505 | rs17686872 | -1290.07 | 72.56 | 6 | 1.22E-13 | 0.0496 | 2594.1 | 2633.0 |
| rs499697 | rs17659389 | rs6702463 | rs17670505 | rs6703014 | -1288.86 | 74.97 | 7 | 1.46E-13 | 0.0512 | 2593.7 | 2638.1 |
| rs7550106 | rs499697 | rs17659389 | rs17670505 | rs3006412 | -1290.28 | 72.13 | 6 | 1.50E-13 | 0.0493 | 2594.5 | 2633.4 |
| rs499697 | rs17659389 | rs17670505 | rs576941 | rs6703014 | -1289.13 | 74.42 | 7 | 1.88E-13 | 0.0509 | 2594.7 | 2638.7 |
| rs7550106 | rs499697 | rs10888501 | rs17659389 | rs17670505 | -1288.07 | 76.55 | 8 | 2.42E-13 | 0.0523 | 2594.1 | 2644.1 |

Abbreviations are as follows: Freq., haplotype frequency; LR, Likelihood ratio; AIC, Akaike information criterion; BIC, Bayesian information criterion.


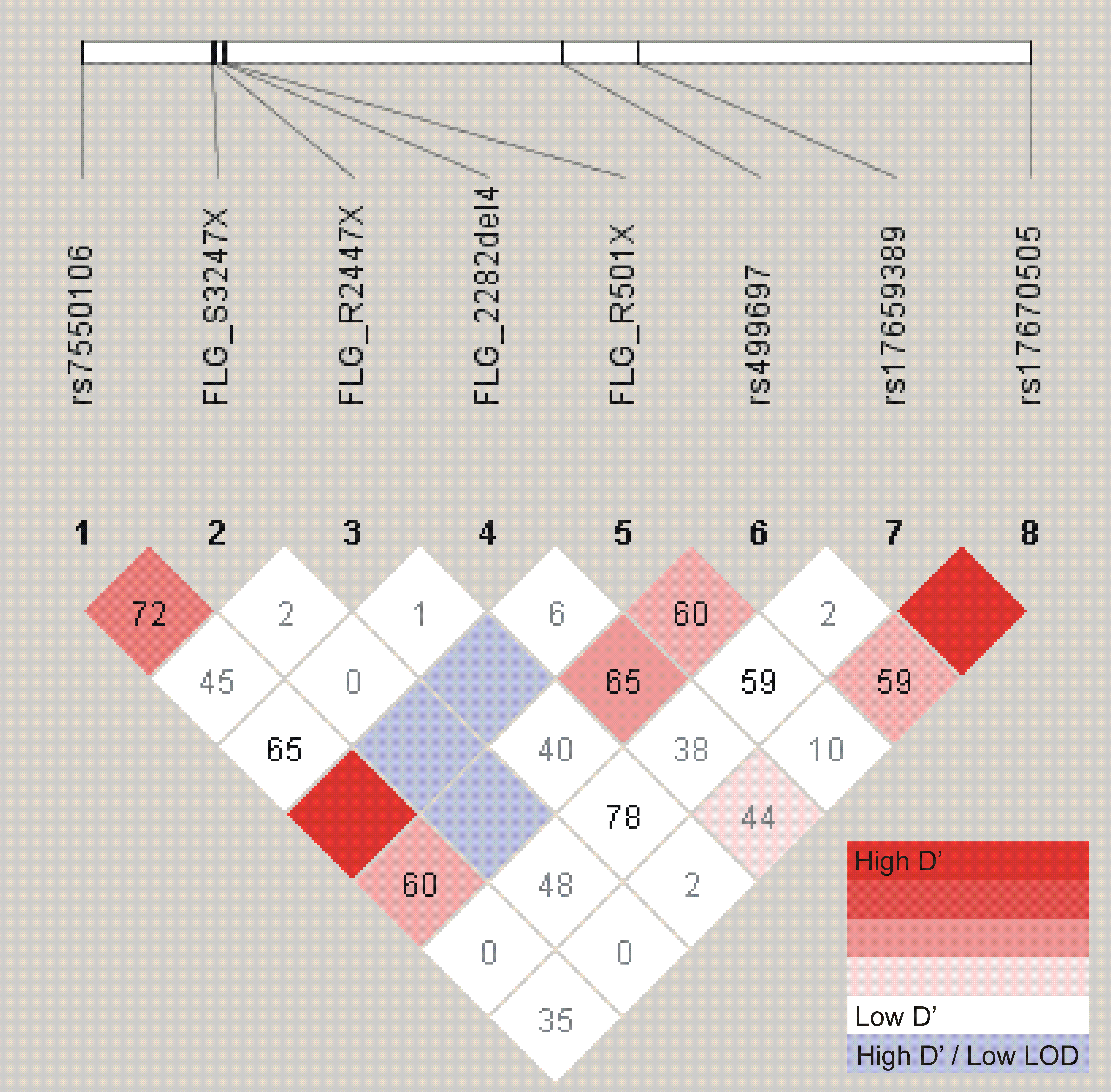


**Figure S1:** LD plot of the four SNPs of the best 4-SNP pattern and the four FLG mutations based on the case control set (order by physical position). LD measure D’ is shown. Created by Haploview 4.2 (Barrett JC et al. Bioinformatics 2005, 21(2):263-265).

**Figure S2**: Mean of –log10(p values) of best 10 p values in each step of the Multi-locus stepwise regression based on the case control sample.
